# *Rhizobium pusense-*Mediated Selenium Nanoparticles–Antibiotics Combinations against *Acanthamoeba* sp.

**DOI:** 10.3390/microorganisms10122502

**Published:** 2022-12-16

**Authors:** Pradnya B. Nikam, Jitendra D. Salunkhe, Kiran R. Marathe, Mousa A. Alghuthaymi, Kamel A. Abd-Elsalam, Satish V. Patil

**Affiliations:** 1 Department of Biochemistry, School of Life Sciences, Kavayitri Bahinabai Chaudhari North Maharashtra University, Jalgaon 425001, India; 2 Biology Department, Science and Humanities College, Shaqra University, Alquwayiyah 11971, Saudi Arabia; 3 Plant Pathology Research Institute, Agricultural Research Center, Giza 12619, Egypt

**Keywords:** *Acanthamoeba*, nano-selenium, trophozoites, cysts, ciprofloxacin, anti-protozoal

## Abstract

Severe ocular infections by *Acanthamoeba* sp. lead to keratitis, resulting in irreversible vision loss in immune-compromised individuals. When a protozoal infection spreads to neural tissues, it causes granulomatous encephalitis, which can be fatal. Treatment often takes longer due to the transition of amoeba from trophozoites to cyst stages, cyst being the dormant form of *Acanthamoeba*. A prolonged use of therapeutic agents, such as ciprofloxacin (Cipro), results in severe side effects; thus, it is critical to improve the therapeutic efficacy of these widely used antibiotics, possibly by limiting the drug-sensitive protozoal-phase transition to cyst formation. Owing to the biomedical potential of selenium nanoparticles (SeNPs), we evaluated the synergistic effects of ciprofloxacin and *Rhizobium pusense*–biogenic SeNPs combination. SeNPs synthesized using *Rhizobium pusense* isolated from root nodules were characterized using UV–Visible spectrophotometer, FT-IR, SEM with EDX, particle size analysis, and Zeta potential. The combination was observed to reduce the sub-lethal dose of Cipro, which may help reduce its side effects. The selenium and ciprofloxacin (SeNPs–Cipro) combination reduced the LC_50_ by 33.43%. The anti-protozoal efficacy of SeNPs–Cipro was found to transduce through decreased protozoal-cyst formations and the inhibition of the galactosidase and protease enzymes of trophozoites. Furthermore, high leakage of sugar, proteins, and amino acids during the SeNPs–Cipro treatment was one primary reason for killing the trophozoites. These experimental results may be helpful in the further pre-clinical evaluation of SeNPs–Cipro to combat protozoal infections. Future studies for combinations of SeNPs with other antibiotics need to be conducted to know the potential of SeNPs against antibiotic resistance in *Acanthamoeba.*

## 1. Introduction

*Acanthamoeba* is a eukaryotic organism found in freshwater and soil. Its cysts are resistant to ultraviolet light, heat, and harsh antibiotics. They feed on other microorganisms such as bacteria, yeast, fungi, and other protozoa. They are opportunistic pathogens and can cause severe problems in favorable conditions, such as *Acanthamoeba* granulomatous encephalitis and *Acanthamoeba* keratitis, caused by *Acanthamoeba castellanii. Acanthamoeba* has a two-stage life cycle, the vegetative growing trophozoite and the dormant cyst stage [1,2,3]. *Acanthamoeba* infections are treated with combinations of various antibiotics and chemotherapeutic agents such as fluconazole (200 mg), neomycin, paromomycin, chlorhexidine (0.02%), hexamidine (0.01%), amphotericin B (varying concentrations), chlorhexidine digluconate (0.02%), and many others in different dosages found to be effective against *Acanthamoeba* infections such as eye keratitis and encephalitis [4,5,6,7,8]. Additionally, high doses, prolonged treatment, poor absorption of drugs, and drug toxicity are major problems in the treatment of *Acanthamoeba* diseases [9]. In this background, there is a need to find effective and safe sources of amoebicidal molecules. In the current era of nanotechnology, various nanomaterials have been reported as therapeutic agents, including antibacterial, anti-fungal, anti-protozoal, etc. Although silver nanoparticles are already reported as anti-*Acanthamoeba* products [10,11,12,13], there is a need to screen other nanomaterials as anti-*Acanthamoeba* drugs due to their toxic effect on human cells [14,15]. In addition, ciprofloxacin, a chemotherapeutic agent, has been previously reported for its anti-*Acanthamoeba* potential. It has been proven to be a significant chemotherapeutic agent against *Acanthamoeba*, and its combinations with voriconazole and chlorohexidine have been found potent in controlling infections [16].

Selenium is recognized as one of the crucial trace elements for biological functioning and its deficiencies may lead to some disorders [17,18]. Various essential proteins, known as seleno-proteins, are also characterized for different roles in mammals, including humans. Nano-selenium, similar to other nanomaterials, has a variety of useful qualities that make it widely used in electronics, agriculture, and the medical field. Compared to bulk selenium, nano-selenium is more bioavailable, and lesstoxic. Selenium nanoparticles (SeNPs) have already been linked to a variety of biological activities, including those that are anti-diabetic, anti-bacterial, anti-fungal, anti-cancer, and anti-oxidative. SeNPs are well known for their drug-delivery capabilities in addition to their extensive therapeutic applications [19,20]. All these reports induced to test its anti-amoebicidal potential. The present study deals with the anti-*Acanthamoeba* capacity of biosynthesized selenium nanoparticles (SeNPs). SeNP combinations with ciprofloxacin were examined for their effectiveness and mode of action in killing both the cyst and trophozoite stages of the *Acanthamoeba* species.

## 2. Material and Methods

### 2.1. Materials

Sodium selenite was purchased from Hi-media (Mumbai) and stored at room temperature. The artificial substrate BAPNA (Benzoyl D-L-Arginine-paranitroanilide) and Triton X- 100 were procured from Sigma chemicals, USA. The standard antibiotic, ciprofloxacin, was purchased from SRL and stored at 4 °C. Ultra-pure grade water was used for all the experiments.

### 2.2. Sample Collection

The bacterial isolates for SeNPs synthesis were collected from the root nodules of black chickpeas (*Cicer arientinum*) from the farms in the Jalgaon district of Maharashtra state. These isolates were grown on yeast extract mannitol salt agar medium and later characterized morphologically, biochemically, and by 16S rRNA sequencing [21]. 

### 2.3. Synthesis of Selenium Nanoparticles (SeNPs)

The 24-hour-old culture was inoculated on a solid nutritional medium containing 2.5 g of yeast extract, 2.5 g of casein enzyme hydrolysate, 1 g of dextrose, and 5 g of sodium chloride per liter of pure distilled water. This medium was supplemented with sterile sodium selenite solution in distilled water (1 mg/mL) after sterilization of the medium. The selenium tolerance and nanoparticle synthesis were investigated by the potential growth and red color change of the colonies. 

The concentration of sodium selenite for the synthesis of nanoparticles was optimized, ranging from 100 to 1000 µg/mL used for the liquid synthesis of nano-selenium by adding the loopful inoculum in the broth with the composition mentioned above. After incubation, the bacterial cells were pelleted out and transferred into the sterile sodium selenite solution to avoid medium-component interference in the biotransformation. The biotransformation was carried out at 37 °C temperature and under continuous shaking at 180 rpm. After complete transformation, the red-colored material was centrifuged (REMI C-24 BL, Mumbai (MH), India) at 18,785 g to remove the leftover selenite traces. The pellets were then subjected to a 10 min ultrasonic treatment, followed by repetitive centrifuge to remove cell debris. The supernatant was collected and used for additional bioassays and characterization. The lyophilized, red, evenly suspended supernatant was gathered and used for additional research and analysis.

### 2.4. Characterization of Selenium Nanoparticles

Different analytical approaches were used to analyze the biosynthesized selenium nanoparticles (SeNPs). One of the key stages to understanding the physiochemical nature of synthesized nanomaterials, which aids in researching their potential uses and toxicity, is their characterization [22]. The lyophilized pure SeNPs were used for further characterization.

The SeNPs synthesized using *Rhizobium* cells suspended in water were confirmed by UV–Vis spectroscopy using the principle of surface plasma resonance. After the incubation of 24 h, the synthesis of SeNPs was confirmed by UV–Vis spectroscopy by taking the spectra at 200–700 nm (Shimadzu 1601, Tokyo, Japan). The FT-IR (Fourier Transmission Infrared Spectroscopy) analysis of the synthesized nano-selenium was performed (Shimadzu FT-IR 8400, Tokyo, Japan) to investigate the role of *Rhizobium* cells in reduction and to cap around SeNPs. The SeNPs samples were scanned in the wavelength range of 400 to 4000 cm^−1^ after blending the dried samples with KBr. 

Field Emission Gun-Scanning electron microscopy (JEOL JSM-7600F FEG-SEM, JEOL USA Inc., Peabody, MA, USA) was carried out to know the structural morphology of the particles. After FEG-SEM, to determine the elemental makeup of that particular region, the electron micrograph was subjected to EDAX (energy-dispersive spectroscopy, Billerica, MA, USA) mapping. By using a Zetasizer by Malvern instrument and a Dynamic Light Scattering instrument (Malvern Panalyticals, Malvern, UK), it was possible to estimate the hydrodynamic diameter and the total charge potential on the manufactured SeNPs.

### 2.5. Amoebicidal Effect

#### 2.5.1. Test Organisms

The pathogenic strain of *A*. *castellanii* (ATCC 50492) was cultured in a PYG (peptone–yeast extract–glucose) medium (2% peptone, 0.2% yeast extract, and 1.8% glucose) at 30 °C. Further, 1 mL of the culture was centrifuged for 5 min at 2000 g (REMI RM 02 plus mini centrifuge, Mumbai, India). After discarding the supernatant, pellets were washed twice with phosphate buffer saline (PBS), followed by diluting it in a PYG medium to obtain its final concentration of 2 × 10^4^ trophozoites per milliliter [10,23].

#### 2.5.2. Axenization of *Acanthamoeba* Cysts

*Acanthamoebae* were axenized by harvesting cysts from the PYG plate cultures by adding 5 mL sterile saline in 2-week-old PYG *Acanthamoeba* seeded plates. This material was centrifuged at 13,149 g (REMI C24 BL centrifuge, Mumbai, India) for 10 min to collect the cysts from the pellet. The pellet was incubated with 3% HCl overnight to eliminate the bacteria. Subsequently, the cysts were washed 2–3 times with saline and again centrifuged at 13,149 g for 10 min to remove the remaining hydrochloric acid, further transferring it to liquid cultures in PYG medium. The culture was maintained in axenic conditions by transferring it to a fresh medium weekly [23].

#### 2.5.3. Amoebicidal Assay

The amoebicidal activity was assessed by adding 100 μL of the culture of *A*. *castellanii* containing 50 trophozoites and 100 μL of each test (SeNPs) concentration at 5, 10 and 50 ppm in sterile polystyrene plates, and plates were sealed followed by incubation at 30 °C. The plates were monitored using an inverted microscope and counted in a Fuchs-Rosenthal counting chamber after 24 h. The trypan blue exclusion method was used to test the cyst viability [11,24]. The negative control consisted of sterile water in the trophozoite suspension. The experiments were performed in triplicate on two different days. The SeNP LC_50_ was first observed using the different test concentrations of SeNPs, i.e., concentrations of 1.56 to 50 ppm were assessed for significant amoebicidal activity. Chlorohexidine was used as a standard anti-*Acanthamoebal* drug (positive control), and its LC_50_ was determined by exposing an increasing concentration of ciprofloxacin solution, i.e., 10 to 100 µg/mL.

#### 2.5.4. Combination Study of SeNPs (Se) and Ciprofloxacin (Cipro)

The following two types of combinations were designed for study:

1. Cipro-Se: The sub-lethal concentration, i.e., the LC_30_ of SeNPs, was combined with increasing ciprofloxacin concentrations, i.e., SeNPs were fixed with varying amounts of ciprofloxacin concentration;
2. Se-Cipro: The sub-lethal concentration of ciprofloxacin was kept constant, which was calculated previously, and the concentration of SeNPs varied from 1.56 to 50 ppm. Both the combinations were exposed to 50 trophozoites in triplicate for 24 h. The LC_50_ value for both combinations was determined by Trypan blue exclusion method.

#### 2.5.5. Mode of Action of Ciprofloxacin and SeNPs Combinations

To determine the mechanism of action on trophozoites, 2 × 10^3^/mL trophozoites in peptone–glucose broth were treated with the LC_30_ concentration of both combinations and diluted up to a final volume of 5 mL of phosphate buffer pH 7 (0.2M) and incubated for 30 min at 30 °C. After centrifugation, the supernatant was checked for various leakages such as sugar, amino acids, and protein. The untreated trophozoites were used as a control. 

#### 2.5.6. Preparations of *Amoeba* Cell Lysates

The freshly cultured 100 trophozoites from PYG agar were collected and added in 5 mL phosphate-buffered saline (pH 7.2) centrifuged and washed with phosphate buffer (pH 7.2). These trophozoites were exposed to sub-lethal concentrations of the SeNPs-Cipro combination and the control (without any treatment), and incubated for 2 h. The cysts were separated from the pellet by centrifuging this material at 13,149 g for 10 min. To get rid of the germs, the pellet was treated with 3% HCl overnight. The cysts were then transferred to liquid cultures in PYG medium after being washed with physiological saline two or three times. The cysts were then centrifuged once more at 13,149 g for 10 min to remove any remaining hydrochloric acid. By switching to a new medium every week, the culture was kept in axenic conditions.

#### 2.5.7. Protease Inhibitor Assay (Trypsin)

Standard Protease (Trypsin) activity was determined by observing the hydrolysis of synthetic substrate BAPNA (Benzoyl D-L-Arginine-paranitroanilide) by measuring the product, i.e., the yellow-color product p-nitroanilidine, at 410 nm. Enzymatic activity is expressed as μM/mg protein/min. Enzyme inhibition as a function of time was determined by trypsin assay in the presence of 100 μL of LC_30_ concentration SeNPs and BAPNA as a substrate for determination of the trypsin inhibitory potential of SeNPs. Additionally, the protein content was quantified by the Bradford method [25].

#### 2.5.8. Encystment Assay

Encystation assays were performed as per previous studies [26,27]. A total of 5 × 10^5^ *Acanthamoeba* trophozoites were incubated with SeNPs and other combinations in a 24-well plate containing PBS and a medium having a composition of 5 mM MgCl_2_ and 8% glucose. These plates were incubated at 30 °C for 72 h. To collect only cysts, 0.25% sodium dodecyl sulfate (SDS) was used. The leftover cysts were counted using a hemocytometer.

#### 2.5.9. Excystation Assay

The excystation assay for *Acanthamoeba castellanii* cysts, was performed according to the method described earlier by Dudley et al. 2009 [28]. A total of 1 × 10^6^ trophozoites were inoculated on non-nutrient agar plates and incubated at 30 °C for 14 days. After this, the cysts were added to 5 mL of PBS buffer saline (PBS) and collected by centrifugation at 3000× *g* for 10 min. These cysts were then suspended in 1 mL of PBS and preserved at 4°C for use in excystation assays after counting. The excystation was measured by incubating 1 × 10^5^ *A. castellanii* cysts with SeNPs at 30 °C. This was observed under the microscope every 24 h for up to 72 h. The emergence of trophozoites was checked and counted by using a hemocytometer.

#### 2.5.10. Toxicity Assay of SeNPs–Cipro Conjugates

The toxicity assays of SeNPs, ciprofloxacin and their conjugate were carried out by lactate dehydrogenase assay by exposing the keratenocytes cell line (HEKa, cell line, American Type Culture Collection, Manassas, VA, USA) to 200 µL cell suspension in each test well at its respective LC_50_ concentration at 24 h in a CO_2_ incubator at 37 °C. This mixture was centrifuged at 400 *g* for 5 min and used for the assay. The supernatant was mixed with 2 µL of sodium lactate, 2 µL of tetrazolium salt and 50 µL of phosphate buffer, incubated at 20 min in the dark, and measured for absorbance at 490 nm. The negative control was PBS buffer, whereas the positive control was 1% triton X-100. All reactions were carried out in triplicate, and the percentage toxicity was calculated using the formula below [29]:

% Toxicity = (OD490 Test)-(OD490 Negative control)/ (OD490 positive control)-(OD490 Negative control)(1)

#### 2.5.11. Statistical Test

The experiments were performed in triplicate and the data presented are in the form of mean ± standard deviation. One-way analysis of variance (ANOVA) was used to analyze the differences in the acquired data. Calculations were done using SPSS software v.20.0.20.0, Armonk, NY, USA. Statistically significant values were considered if *p* ≤ 0.05.

## 3. Results 

### 3.1. Synthesis and Characterization of Selenium Nanoparticles

**Figure 1:**
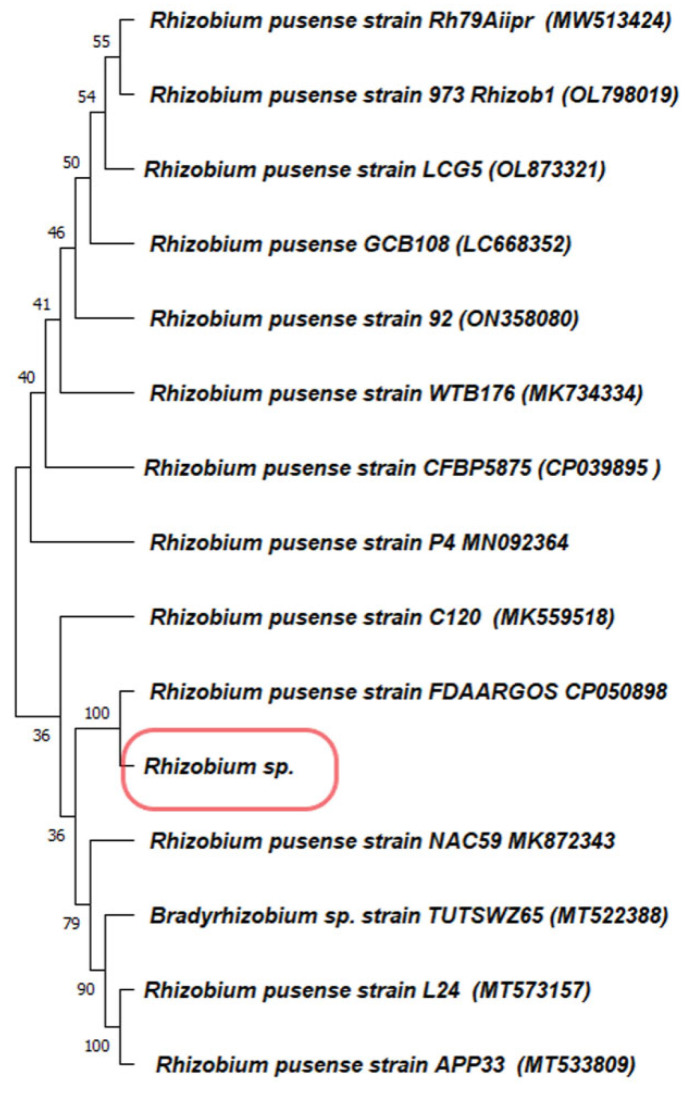
Phylogenetic tree of the bacterial isolate from the root nodules.

The isolated bacteria from the root nodules were gram-negative and motile. They produced oxidase, catalase, and indole, whereas methyl red, Vogus-Prausker, and citrate utilization tested negative. The 16S rRNA gene sequencing confirmed that the bacteria were *Rhizobium pusense* with 100% similarity. The phylogenetic tree was designed by using the MEGA software v.11 (Figure 1). This isolate was able to tolerate about 1000 µg/mL sodium selenite. The transformation of selenite into red nano-selenium was observed in both solid and liquid medium after the incubation period. The color intensity increased with respect to the increase in incubation time. The SeNPs synthesized in the liquid medium were separated using centrifugation followed by sonication to separate them from the cellular material. 

**Figure 2:**
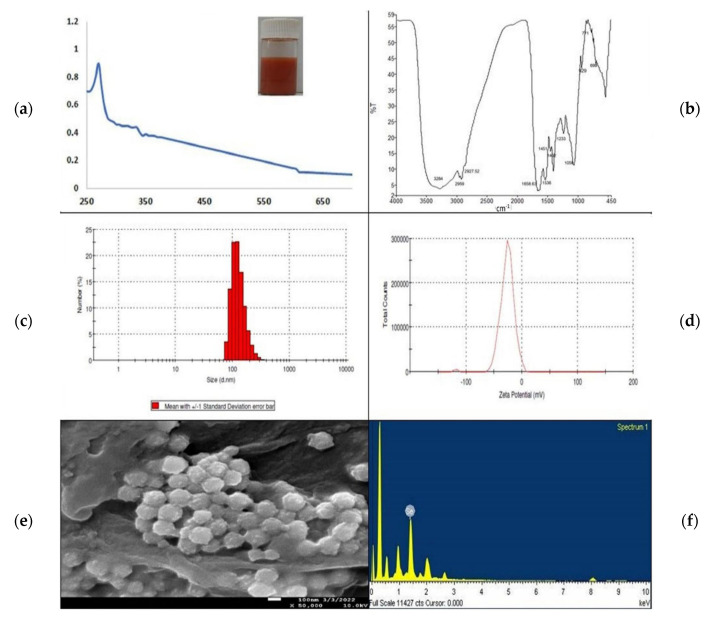
Characterization of selenium nanoparticles; (**a**) UV–Visible spectrum; (**b**) FTIR spectrum; (**c**) Particle size analysis graph; (**d**) Zeta potential; (**e**) FEG-SEM image; (**f**) EDAX spectrum.

The red-colored suspended material was used for characterization using several methods, including UV–visible spectrophotometry, FTIR spectrophotometry, DLS, SEM, and TEM. The scanning of the red colloidal sample of the SeNPs using a UV–Visible spectrophotometer in the range 200–800 nm showed a sharp spectrum at 269 nm due to the Surface Plasmon Resonance mechanism [10] (Figure 2a). In Figure 2b, the FTIR spectrum gives away important functional groups responsible for the biotransformation of selenite into SeNPs. The intense broad peak at 3284 cm^−1^ belongs to the –OH stretch of the hydroxyl group, whereas the small outgrowths at 2959 and 2927 cm^−1^ are for the –C–H stretch of the methyl and methylene groups, respectively. The peak at 1658 cm^−1^ shows alkenyl C=C stretching. The aromatic nitro compounds can be detected because of the peak at 1536 cm^−1^. Peaks at 1451 cm^−1^ are attributed to carbonate ions, and at 1402 cm^−1^, to –OH bending because of the phenol or tertiary alcohol. The peak values 1233 cm^−1^ and 929 cm^−1^ refer to the P–O–P stretching of aromatic phosphates, while other peaks such as 1059 represent phosphate ions. The peak at 771 cm^−1^ represents skeletal C-C vibrations in methylene, and the one at 699 cm^−1^ represents C–H bending of the aromatic group. Therefore, we can say that these complex functional groups contribute to the synthesis and stabilization of the biogenic SeNPs [30,31].

The particle size of the biosynthesized SeNPs ranged from 78–190 nm, and the average particle size was found to be 172.8 nm, having a polydispersity index of 0.066 (Figure 2c), which corresponds to the homogeneity and fine dispersion with the most negligible aggregation of the nanoparticles [31,32]. Along with this, the zeta potential (Figure 2d) at −26.9 indicates good stability of the biogenic SeNPs. The FEG-SEM micrograph has the spherical shape of SeNPs with an average size between 90 and 170 nm (Figure 2e). The EDAX graph for the FEG-SEM image has the characteristic peak for selenium (Figure 2f). All these outcomes confirmed the biosynthesis of spherical and stable SeNPs.

### 3.2. Anti-Acanthamoeba Activity

**Figure 3:**
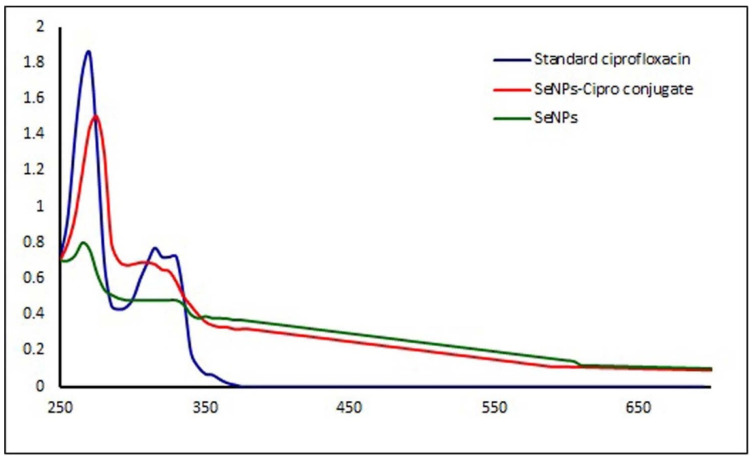
UV–Visible spectra of the combinations used for the experiment.

Selenium nanoparticles have been extensively reported for their antibacterial, antifungal, anti-viral, and insecticidal applications [33,34]. For the first time, the anti-*Acanthamoeba* activity of these SeNPs has been studied by using the *Rhizobium* sp. For this, the lyophilized selenium nanomaterial was suspended in a phosphate buffer containing 0.01% starch, and different concentrations from 1.56 to 50 ppmwere tested against fifty trophozoites of *Acanthamoeba* and incubated for 48 h. It was observed that the SeNPs showed 50% mortality at a very high dose (LC_50_ at 72 ppm) against the tested trophozoites. As Se nanoparticles are well recognized as a vehicle for drug delivery [9], and ciprofloxacin has been reported as an effective drug for *Acanthamoeba* keratitis [20], the combination study of both components was designed. The conformation of the conjugate formation of SeNPs and ciprofloxacin was confirmed using a UV–Visible spectrophotometer (Shimadzu 1601, Tokyo, Japan). Standard ciprofloxacin had maximum absorbance at 271 nm [34], SeNPs at 269 nm, and the combination of SeNPsand ciprofloxacin had their maximum absorbance peak at 276 nm. This shifting of wavelength for the combination shows that SeNPs developed a conjugate with ciprofloxacin, which may be responsible for enhancing its activity. Such results have been reported in the case of gold nanoparticles conjugated with different antibiotics (Figure 3) [35].

Primarily, the LC_50_ of ciprofloxacin against the trophozoites was determined and found to be 56.260 ppm after 48 h. Ciprofloxacin is a standard drug, and it showed a comparatively lower LC_50_ value than SeNPs. Hence, in the first combination, i.e., LC_30_ of SeNPs, the concentration was combined with different concentrations of ciprofloxacin and tested against the trophozoites. However, the Cipro–Se conjugation did not show any significant increase in amoebicidal activity, i.e., the LC_50_ was reduced by only 4.19%. In this background, the second combination, i.e., Se–Cipro, was tested against the trophozoites. The Se–Cipro combination showed a significant decrease in the LC_50_.The LC_50_ was recorded at 37.452 ppm concentration of Se–Cipro for *Acanthamoeba*, reducing the dose by 34.5% (Table 1) compared to the ciprofloxacin treatment.

### 3.3. The Cysticidal Effect of SeNPs and SeNP–Cipro (Se–Cipro)

SeNPs and Se-Cipro combinations tested for encystment and excystment effect on *Acanthamoebae* trophozoites showed significant mortality. Among all the given treatments, SeNPs allowed maximum cyst formation of the treated trophozoites (22.066% encystment), while Cipro–Se and ciprofloxacin allowed only 7% and 10% of encystment of the treated trophozoites, respectively (Table 2). The Se–Cipro, which proved to be an effective anti-*Acanthamoeba* preparation, also allowed less encystment (4.66%) of the treated trophozoites. The Se-Cipro treatment significantly inhibited the excystment process of the cysts and this inhibition was constant even after the addition of fresh culture medium into it. 

**Figure 4:**
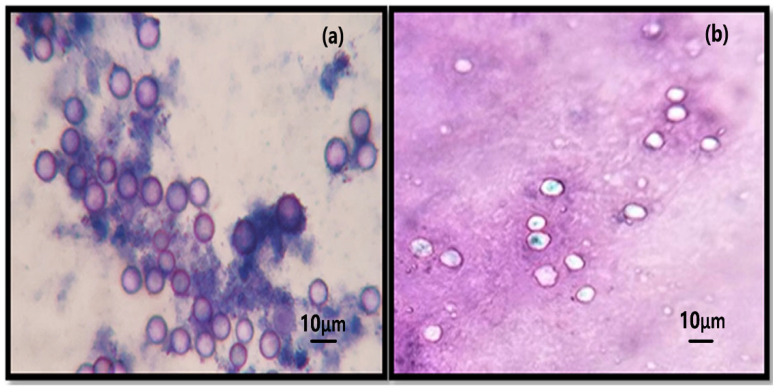
Cysticidal activity of SeNPs–Cipro conjugates: (**a**) Cyst treated with SeNPs–Cipro conjugate; (**b**) control cyst excludes trypan blue.

The trypan blue exclusion assay was performed to check live and killed cysts after every treatment to the cysts using a 40X magnification of the light microscope. The live cysts exclude the stain due to their respiratory mechanism, whereas the killed cysts remain blue as they lose the ability to exclude the stain (Figure 4) [36].

### 3.4. Mechanism of Anti-Acanthamoeba Activity of SeNPs

**Figure 5:**
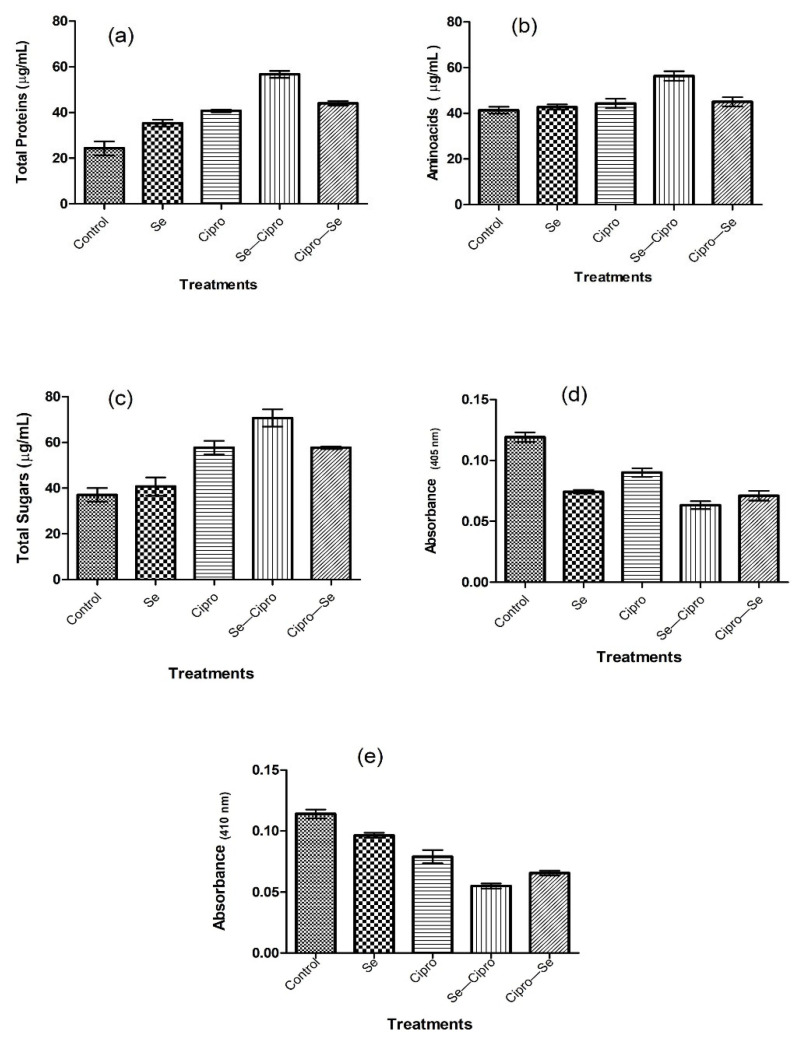
Graphs of bioassays after the different treatments of selenium nanoparticles (Se) and ciprofloxacin (Cipro) given to *Acanthamoeba* trophozoites; (**a**) Protein leakage; (**b**) Amino acid leakage; (**c**) Total sugar leakage; (**d**) Galactosidase inhibitions; (**e**) Protease inhibition.

Similarly to earlier-reported drugs such as chlorohexidine [37,38], the SeNPs synthesized using the *Rhizobium* sp. and their combination with ciprofloxacin were found to have the same mode of action against the *Acanthamoeba* trophozoites, i.e., a significant leakage of essential proteins, enzymes, amino acids, and sugars. Microbial synthesized SeNPs were found to have significant anti-*Acanthamoeba* potential. As the membrane is the primary target for anti-protozoal drugs [39], the effect of selenium nanoparticles, standard antibiotics, and the combinations of both were studied on *Acanthamoeba* cells by analyzing the leakage assays. *Acanthamoeba* cells were treated with the LC_30_ of all the tested material for 3 h, and the broth was analyzed for the leakage of amino acids, proteins, and sugars. It was revealed that the Se–Cipro combination causes significant leakage of the protozoal membrane, indicating damage to cellular integrity as a potential mechanism of action of the combinations. There was significant protein, amino acid, and sugar leakage as vital nutrients compared to the control. There was no significant inhibition of enzyme action after the SeNPs treatment; however, Se–Cipro efficiently inhibited the proteases of *Acanthamoeba* lysates. As is the case with proteases, β-galactosidase is a vital enzyme of *Acanthamoeba* that plays an essential role in *Acanthamoeba*’s life cycle and pathogenesis. The Se–Cipro composite was also found to be a significant inhibitor of β-galactosidase of the *Acanthamoeba* cells, i.e., the lysates (Figure 5).

### 3.5. Toxicity Testing

**Figure 6:**
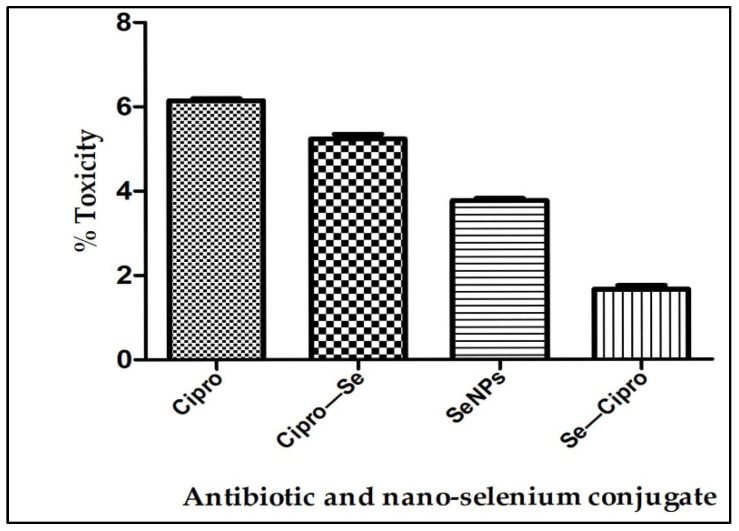
Graphical representation of the percentage toxicity of the different treatments given to the *Acanthamoeba* cells.

The toxicity of all the compounds tested against the *Acanthamoeba* species was evaluated fortheir non-targeted toxicity against the keratinocytes cell line (HEKa, cell line). The study revealed that the LC_50_ value of the Se–Cipro nanoconjugate was non-toxic and the percentage toxicity calculated was 1.792%. The ciprofloxacin at its LC_50_ concentration showed a toxicity of 6.1%, but after conjugation, it was reduced. The SeNPs used were also found non-toxic at its LC_50_ concentrations. Overall, the study reveals the synthesized Se–Cipro conjugates were non-toxic (Figure 6).

## 4. Discussion

The management of diseases caused by *Acanthamoeba*, particularly amoebic keratitis (AK), is very challenging for healthcare providers. The available therapeutic medications for the same have drawbacks such as prolonged treatment, and require the combination of multiple drug courses [40]. Hence, to combat ocular protozoa, it is essential to increase the efficacy of pre-existing antibiotics. Nanoparticle-based formulations have an effective drug-delivery system against the *Acanthamoeba* species due to their easy accessibility to the infected organ, the release of drugs at a high concentration to the target site, and the increase in drug solubility and stability [41]. Biocompatible SeNPs (selenium nanoparticles) are well known for their drug delivery to specific cells and their increasing drug efficacy [42,43]. To access an increase in the amoebicidal efficacy of the SeNPs–Cipro combination, we first aimed to biosynthesize SeNPs using the *Rhizobium* species isolated from the root nodules of *Cicer arientinum.* Therefore, first, we isolated gram-negative and motile bacteria from the root nodules. The biochemical test and 16S rRNA sequencing confirmed the isolate as *Rhizobium pusense*. This microbial isolate was able to produce dark-red colonies, which primarily indicates that the organism highly tolerates selenium and produces nano-selenium. The intense red-color formation indicates selenite was converted in the nanomaterial, similar to a previous report for the *Pseudomonas* sp. [44]. The bacterial isolate also transformed selenite into red nano-selenium in the liquid medium after an incubation of 24 h. It was observed that the accumulation of nanomaterial takes place both intra- and extracellularly; upon cell lysis, the SeNPs are released into the solution. SeNPs were separated using centrifugation followed by sonication. This red-colored suspended material showed a sharp spectrum at 269 nm when observed under a UV–Visible spectrophotometer. Earlier studies on synthesizing SeNPs using ethanolic extract of bee propolis and lysozyme-fabricated SeNPs have also remarked the spectrum of selenium nanoparticles at 265 nm [45,46]. The spectrum obtained is due to the surface plasmon resonance of the nanoparticles in the suspension. There are also some other shreds of evidence that manifest the SeNPs spectrum at different wavelengths. SeNPs synthesized using *Fusarium oxysporum* had the maximum absorbance at 217 nm [30]; *Emblica officinalis* fruit extract had the SeNPs spectrum at 270 nm [32]; SeNPs synthesized by the seed powder of *Mucuna pruriens* at 319 nm [31]; additionally, in many more reports, UV–Visible spectrum showed slight variations. The FTIR spectra of *Rhizobium*-mediated synthesized SeNPs confirm the presence of complex functional groups that contribute to the synthesis and stabilization of the biogenic SeNPs [30,47]. The FEG-SEM micrograph of selenium nanoparticles has a spherical shape with an average size between 90 and 170 nm, which also confirmed the biosynthesis of SeNPs. 

Currently available multipurpose contact-lens disinfection systems are not fully effective against *Acanthamoeba* trophozoites and cysts. It was observed that even after treatment with strong amoebicidal drugs such as chlorohexidine and polyhexamethylene biguanide, *Acanthamoeba* infections persist as active infections. Hence, there is an urge to increase the disinfecting activity of these systems to prevent *Acanthamoeba keratitis* infections [48]. Niyyati et al. (2018) also advocated using gold and silver nanoparticles as lens disinfectants to avoid *Acanthamoeba* infections [49].

SeNPs have been mostly reported as potential antibacterial, antifungal, anti-viral and insecticidal agents [20,33]. The anti-*Acanthamoebal* activity of various nanoparticles such as silver, gold, cobalt, and zinc have been extensively studied [41], but there are no reports on selenium nanoparticles. However, the present study provides the basis for further applications of selenium nanoconjugates as anti-*Acanthamoebal* agents. In the present study, we have tested antibiotics and selenium-nanoparticle conjugates against the human pathogen *Acanthamoeba castellanii*. It was observed that there is a significant decrease in the LC_50_ value of the Se–Cipro nanoconjugates (34.5%) compared to ciprofloxacin [50,51]. The hype in the anti-*Acanthamoeba* activity of antibiotics and nano-selenium conjugates compared to basic compounds is similar to our previous reports of Salunkhe et al. (2022), who advocated that the naringenin–nano-silver conjugates reduced the LC_50_ of naringenin by 50.56% [52]. Similarly, Padzik et al. (2018) also observed that the conjugated nanomaterial increases the activity against *Acanthamoeba* keratitis [53]. The rise in anti-*Acanthamoebal* potential tannic acid–silver nanoparticle conjugates (AgTANPs) may be due to their high absorption in amoebic cells. Aqeel et al. (2016) reported that gold nanomaterial conjugates with biguanides, such as chlorhexidine digluconate, and other drugs are more effective against the *Acanthamoeba* sp. than the standard drug [54]. The two major modes of action for the Se–Cipro conjugate tested were found to be the leakage of membrane and the inhibition of essential enzymes such us trypsin in the amoebic cells. It was observed that the *Acanthamoeba* cells treated with the LC_30_ for 3 h by the Se–Cipro conjugate caused the leakage of amino acids, proteins, and sugars. Hence, it was revealed that the damage in cellular integrity is an indication of the potential mechanism of the drug and SeNPs combinations as compared to standard ciprofloxacin. Xuguang et al. (2003) advocated the same mode of action, i.e., disruptions of the *Acanthamoebal* membrane by the major available chemotherapeutic drugs [55]. Beside these, Se–Cipro significantly inhibits crude enzymes such as the proteases of *Acanthamoeba* lysates. In addition to the proteases, Se–Cipro significantly inhibited β-galactosidase, which is a vital enzyme required for completing the life cycles of *Acanthamoeba* [56]. Many reports suggest that enzymatic activities, including cytolytic mechanisms and vacuole digestions, may play a significant role in the life cycle and metabolism of trophozoites. Amoebae produce a variety of proteases that can participate in the damage of the corneal tissue. Amoebic proteolytic enzymes include serine proteases [57,58], contact-dependent metalloproteases [59], elastases [60], cysteine proteases [57], and mannose-mediated adhesion-induced cytotoxic proteases [61].

All these tested materials were checked for their toxicity on human keratinocytes by LDH assay. The results show that the positive control, i.e., 1% triton X-100, shows 95% toxicity, ciprofloxacin shows 6.1%, whereas the cells without any treatment (negative control) had 2% toxicity, and the Se–Cipro shows 1.79% toxicity. A lower level of LDH corresponds to less cellular disruption [29] and, hence, it indicates that the Se–Cipro combination has negligible toxicity on human cells.

## 5. Conclusions

The *Acanthamoeba* sp. is well known for its opportunistic pathogenesis in human diseases such as an eye infection i.e. Acanthamoeba keratitisand Acanthamoeba granulomatous encephalitis. Although the development of various drugs and supportive therapies is available, *Acanthamoeba* infections are still a major public health concern. Currently available drugs are not fully effective against the trophozoite and cyst forms of *Acanthamoeba*. Although various drugs, nanomaterials, and their conjugates, e.g., cobalt, tannic acid, silver, etc., have been reported for their anti-*Acanthamoeba* activity, there are some toxicity concerns regarding these metal conjugates, due to the generation of highly reactive oxygen species [48,49]. For this reason, the current selenium–antibiotic combination is a safe formulation, as selenium is well known for its antioxidant potential. In an epitome, we biosynthesized NPs using *Rhizobium pusense* isolated from the rhizosphere. The biosynthesized SeNPs were observed to have anti-*Acanthamoeba* activity. The sub-lethal concentration of SeNPs enhanced the anti-protozoal activity of ciprofloxacin. The combination also effectively limited the *Acanthamoeba* phase maturation to dormancy, which can increase the protozoal sensitivity towards antibiotics. The anti-protozoal action of the combo transduces through the destabilization of the protozoal membrane and the inhibition of essential enzymes. The toxicity assay, i.e., LDH, proved that the LC_50_ and LD_90_ concentrations of selenium and Se–Cipro conjugate are comparatively non-toxic, indicating the proposed conjugate may be a future anti-*Acanthamoeba* drug. The experimental observations of the study demand further preclinical studies for using SeNPs and their combination with antibiotics as potential amoebicidal agents.

## 6. Future Perspectives

The studies conducted were limited to using combinations of ciprofloxacin with biogenic SeNPs against the protozoan *Acanthamoeba*. More investigations on the combinational studies of SeNPs with other antibiotics, natural products, and other nanomaterials to combat antibiotic-resistance in the *Acanthamoeba* species need to be carried out in the future.

## Figures and Tables

**Table 1:** Anti-*Acanthamoeba* activity of nano-selenium (Se), ciprofloxacin, and their conjugate.

Test Species	Test Products	LC_50_ ± SD (mg lit^–1^)	95% Fiducial Limits	LC_90_ ± SD (mg lit^–1^)	95% Fiducial Limits	Regression Equation
*Acanthamoeba* sp.	SeNPs	72.084 ± 10.87	56.593–106.58	115.058 ± 19.264	87.7664–176.491	Y = 0.423 + 0.149X
	Cipro	56.2608 ± 2.2078	51.7279–60.43	125.0330 ± 3.13	119.2960–131.69	Y = 9.27 + 0.274 X
	Cipro–Se	53.9078 ± 5.4802	45.4017–68.6011	94.1228 ± 11.056	77.1133–124.0217	Y = 1.55 + 0.302 X
	Se–Cipro	37.452 ± 3.31	32.196–46.153	94.122 ± 11.05	77.113–124.021	Y= 4.78 + 0.980 X
	Sodium selenite	120.143± 2.56	115.316- 125.444	205.1626± 6.389	193.82–219.166	Y= 8.53 + 0.960 X
	Chlorohexidine	24.580 ± 1.25	22.361- 27.36	56.070 ± 3.61	49.95–64.52	Y= 6.52 + 0.754 X

Level of significance: *p* < 0.05; Y—mortality rate (significant at *p* < 0.05 level); X—concentration (significant at *p* < 0.05 level); LC_50_—lethal concentration that kills 50% of the exposed trophozoites; LC_90_—lethal concentration that kills 90% of the exposed trophozoites; SE—standard error (all values are mean of four replicates); Cipro–Se—varied concentration of Cipro with constant sub-lethal concentration of Se; Se–Cipro—varied concentration of Se with constant sub-lethal concentration of Cipro; Chlorohexidine—a standard anti-*Acanthamoeba* drug (positive control). The LC_50_ and LC_90_ concentration units are given in mg lit^−1^, which also mean as ppm.

**Table 2:** Encystment and excystment at LC_50_ concentration of nano-combinations and control.

Sr. No.	Concentrations ppm	Total No. of Cysts	No. Cyst After 24 hrs.	% Encystment	% Excystment
1	56.260 Cipro	50	5	10.66	Nil
2	72.084 Se	50	10	22.066	Nil
3	53.907 Cipro–Se	50	3	7.33	Nil
4	37.452 Se–Cipro	50	2	4.66	Nil
5	Control	50	45		39.33

## Data Availability

Not applicable.
